# Identification of Pathogenicity-Associated Loci in Klebsiella pneumoniae from Hospitalized Patients

**DOI:** 10.1128/mSystems.00015-18

**Published:** 2018-06-26

**Authors:** Rebekah M. Martin, Jie Cao, Weisheng Wu, Lili Zhao, David M. Manthei, Ali Pirani, Evan Snitkin, Preeti N. Malani, Krishna Rao, Michael A. Bachman

**Affiliations:** a Department of Pathology, Michigan Medicine, Ann Arbor, Michigan, USA; b Department of Biostatistics, School of Public Health, University of Michigan, Ann Arbor, Michigan, USA; c BRCF Bioinformatics Core, University of Michigan, Ann Arbor, Michigan, USA; d Department of Microbiology and Immunology, Michigan Medicine, Ann Arbor, Michigan, USA; e Division of Infectious Diseases, Department of Internal Medicine, Michigan Medicine, Ann Arbor, Michigan, USA; Duke University Medical Center

**Keywords:** *Klebsiella*, comparative genomics, intestinal colonization, pathogenesis, psicose, tellurite

## Abstract

Despite insights gained through experimental models, the set of bacterial genes important for human infection is unclear for many of our most threatening pathogens. Klebsiella pneumoniae is a leading cause of health care-associated infections (HAIs) and commonly colonizes hospitalized patients, but the factors that determine whether a particular isolate causes disease or remains a colonizer are poorly understood. To identify bacterial genes associated with K. pneumoniae infection, a case-control study was performed comparing infected and asymptomatic colonized patients. Comparative bacterial genomics was combined with a conditional logit model that identified patient factors differentiating cases from controls. This method identified five gene loci associated with infection after adjustment for patient factors, including a psicose sugar utilization locus that was validated as a fitness factor during mouse lung infection. These results indicate that bacterial genome-wide association studies of patients can identify loci associated with HAIs and important in infection models.

**IMPORTANCE** Klebsiella pneumoniae is a common cause of infections in the health care setting. This work supports a paradigm for K. pneumoniae pathogenesis where the accessory genome, composed of genes present in some but not all isolates, influences whether a strain causes infection or asymptomatic colonization, after accounting for patient-level factors. Identification of patients at high risk of infection could allow interventions to prevent or rapidly treat K. pneumoniae infections.

**Author Video**: An author video summary of this article is available.

## Abstract (precis)

Klebsiella pneumoniae is a common cause of infections in the health care setting. This work supports a paradigm for K. pneumoniae pathogenesis where the accessory genome, composed of genes present in some but not all isolates, influences whether a strain causes infection or asymptomatic colonization, after accounting for patient-level factors. Identification of patients at high risk of infection could allow interventions to prevent or rapidly treat K. pneumoniae infections.

## INTRODUCTION

Klebsiella pneumoniae is a Gram-negative bacterial pathogen and a leading cause of health care-associated infections (HAIs) in the United States (1). In addition, hypervirulent community-associated (CA) strains that infect seemingly healthy individuals have emerged. These strains are particularly worrisome since they cause severe diseases such as pyogenic liver abscess (PLA), meningitis, and endophthalmitis (2–4). Originating in the Asian Pacific Rim, these strains are now being reported globally, including in the United States (5–7). Similarly, antibiotic resistance in K. pneumoniae has become an emerging problem. In fact, in 2013, the Centers for Disease Control and Prevention (CDC) designated carbapenem-resistant *Enterobacteriaceae* (CRE), of which *Klebsiella* species are the most prevalent, as an urgent threat (8). Further complicating the treatment of these infections is that approximately 20% of isolates identified as K. pneumoniae are not actually K. pneumoniae species (Kp phylogroup I), but are either Klebsiella quasipneumoniae (Kp phylogroup II) or Klebsiella variicola (Kp phylogroup III) (9–12). About 50% of K. quasipneumoniae are extended-spectrum beta-lactamase (ESBL) producers (13), and K. variicola has been associated with higher mortality in bloodstream infections (9). Therefore, correct identification of Kp phylogroups may impact patient care. Together, K. pneumoniae and closely related species represent a significant health care burden.

K. pneumoniae commonly colonizes the nasopharynxes and gastrointestinal tracts of hospitalized patients (14). In a cohort study of hospitalized patients, intestinal colonization with K. pneumoniae was significantly and independently associated with infection with K. pneumoniae, and patients were frequently infected with the same strain they were colonized with (15). This association was independently confirmed (16) and suggests colonization as a reservoir for infection, and thus as an important step in the pathogenesis of K. pneumoniae infection. Infections caused by these bacteria include pneumonia, septicemia, urinary tract infections, and wound infections. In an effort to understand the broad pathogenesis determinants for K. pneumoniae, several virulence genes have been identified. These include genes involved in evasion of complement, use of siderophores for iron acquisition, and genes encoding capsule or adhesins (14). However, a subset of colonized patients do not proceed to disease, instead remaining asymptomatically colonized. The bacterial factors that determine whether an isolate causes disease or remains a colonizer are poorly understood.

Increasing capabilities of whole-genome sequencing (WGS) and comparative genomics allow for effective identification of virulence factors in bacteria, including K. pneumoniae (13). These techniques can delineate the bacterial accessory genome, which varies among isolates, from the conserved core genome and have identified genes that aid in virulence of uropathogenic Escherichia coli (UPEC) (17) and K. pneumoniae (13). This opens the door for genome-wide association studies (GWAS) to compare strains that do or do not cause clinical disease. GWAS have been performed successfully with human genomes to identify genetic variants significantly associated with particular diseases. Recently, bacterial GWAS have identified associations between bacterial polymorphisms or novel loci and specific bacterial phenotypes (18–21).

This study combined bacterial GWAS and clinical modeling to identify patient and bacterial factors associated with K. pneumoniae infection compared to asymptomatic colonization. We hypothesized that genes in the accessory genome of K. pneumoniae promote extraintestinal infection. To test this hypothesis, we performed a case-control study with 1:2 matching and analyzed the gene frequency differences between bacterial isolates using a novel comparative genomics technique we termed pathogenicity-associated locus sequencing (PAL-Seq). We then assessed whether candidate virulence genes were independent predictors of infection, whether they improved prediction of infection when incorporated into a clinical model, and whether they had a distinct phenotype in a murine model of pneumonia.

## RESULTS

### Study design and patient demographics.

During a 3-month period from 30 July 2014 to 31 October 2014, more than 2,000 patients (age range, 16 to 89 years) were screened in intensive care and hematology/oncology units for K. pneumoniae colonization by rectal swab culture. Simultaneously, patients were screened hospital-wide for extraintestinal infection with K. pneumoniae based on positive clinical cultures from blood or respiratory tract. We identified 38 patients meeting case definitions for extraintestinal infection, either bacteremia or pneumonia (22). One patient had two isolates separated in time that met the case definition; only the first invasive infection from this patient was counted for the case-control analysis. However, all isolates were included for sequencing. Each case patient (*n* = 38) was matched to two asymptomatically colonized controls (*n* = 76) based on age range (within 10 years), gender, and sample collection date (within 3 weeks).

### Identification of patient variables differentiating cases from controls.

**FIG 1:**
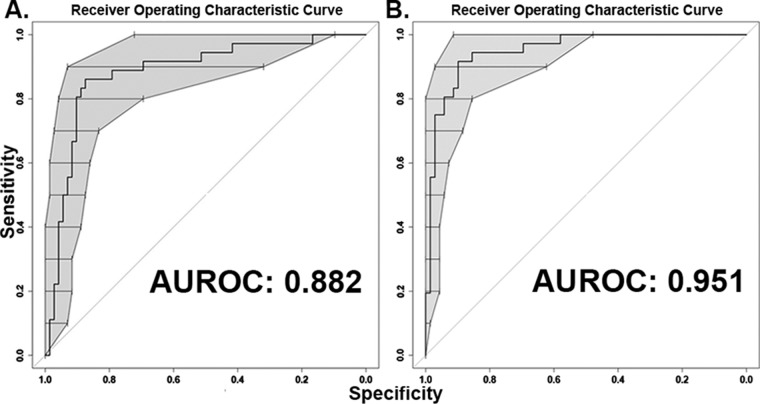
Receiver operating characteristic curve for multivariable models of factors predictive for patient infection. (A) Conditional logistic regression of patient factors was used to generate a predictive model (area under the receiver operating characteristic curve [AUROC] of 0.88). Six clinical variables associated with invasive infection in this sample set. (B) Conditional logistic regression identified bacterial genes that are associated with disease and improved AUROC to 0.95 when added to the clinical model (*P* = 0.011). Central line was removed in the final model. The bars and shaded area of ROC curve in panels A and B represent bootstrapped 95% confidence intervals for specificity at each level of sensitivity.

To determine whether there were any significant differences in clinical characteristics between cases (*n* = 36) and controls (*n* = 72), bivariable analysis was performed (Table 1). Two case patients were excluded from this analysis, along with their corresponding controls, since the patient data for these cases were unreliable or unavailable. Only white race showed a significant difference (66.7% versus 84.7%; *P* = 0.033), with an inverse association with infection. A multivariable model constructed using backwards elimination included six patient variables and differentiated the cases from the controls in this cohort (Table 2). The model’s area under the receiver operating characteristic curve (AUROC) was 0.88 (Fig. 1A). Interactions among these variables in the final model were tested, and none were significant. In this model, the presence of a central venous catheter was not associated with infection (*P* = 0.075 when adjusted for covariates in the model) but was retained as it contributed significantly to model fit by likelihood ratio testing, though this was borderline (*P* = 0.054).

**TABLE 1:** Demographic and clinical characteristics of case and control patients

Characteristic	No. of patients (%) or parameter value (mean ± SD)		*P* value (logit)
	Case^a^ (*n* = 36)	Control^b^ (*n* = 72)	
Age (yr)	61.5 ± 18.5	61.6 ± 17	0.857
Female	18 (50)	36 (50)	>0.99
White race	24 (66.7)	61 (84.7)	0.033
Admitted from nursing home	1 (2.8)	1 (1.4)	0.624
White blood cell count (baseline) (in thousands per μl)	10 ± 6.2	16.5 ± 28.4	0.138
Hemoglobin level (baseline) (g/dl)	10.3 ± 2.2	10.7 ± 2.3	0.354
Platelet count (baseline) (in thousands per μl)	177.8 ± 90.8	185.1 ± 112.5	0.798
Creatinine level (baseline) (mg/dl)	1.2 ± 1.6	1.4 ± 1.7	0.599
Albumin level (baseline) (g/dl)	3.4 ± 0.57	3.3 ± 0.64	0.615
Glucose level (mg/dl)			
Baseline	137.9 ± 59.3	131.2 ± 55.7	0.572
Minimum	93.3 ± 21.5	86.9 ± 21.4	0.181
Maximum	209.8 ± 101.2	193.1 ± 85.3	0.337
Body mass index (baseline) (kg/m^2^)	25.2 ± 8	30.5 ± 8.5	0.134
Central venous catheter	13 (36.1)	36 (50)	0.17
Fluid and electrolyte disorders	21 (58.3)	32 (44.4)	0.193
Peripheral vascular disease	1 (2.8)	8 (11.1)	0.191
Other neurological disorders	4 (11.1)	1 (1.4)	0.063
Pulmonary disorders	5 (13.9)	8 (11.1)	0.684
Diabetes without chronic complications	8 (22.2)	11 (15.3)	0.506
Renal failure	5 (13.9)	13 (18.1)	0.572
Liver disease	1 (2.8)	8 (11.1)	0.166
Lymphoma	2 (5.6)	4 (5.6)	>0.99
Metastatic cancer	3 (8.3)	3 (4.2)	0.355
Solid tumor without metastasis	9 (25)	18 (25)	>0.99
Alcohol abuse	0 (0)	5 (6.9)	>0.99
Anemia deficiency	10 (27.8)	16 (22.2)	0.538

a Patients with bloodstream infection or pneumonia.

b Patients with asymptomatic rectal colonization.

**TABLE 2:** Multivariable model for clinical infection with K. pneumoniae^a^

Patient variable or bacterial gene	Clinical model			Final model		
	OR	CI	*P*	OR	CI	*P*
Patient variables						
Fluid and electrolyte disorders	5.1	1.29–20.17	0.02	22.9	1.6–329	0.021
Minimum serum glucose level (mg/dl)	1.04	1.01–1.08	0.009	1.08	1.01–1.16	0.026
Body mass index (baseline) (kg/m^2^)	0.86	0.77–0.97	0.011	0.69	0.5–0.95	0.022
White race	0.13	0.03–0.59	0.008	0.03	<0.01–0.75	0.032
Peripheral vascular disease	0.03	0.001–0.67	0.027	0.001	<0.01–3,290	0.376
Central venous catheter	0.29	0.08–1.13	0.075			
Bacterial genes						
Tellurium resistance protein TerF (KP1_RS26720)	11.3^b^	1.6–80	0.015	157^c^	3.34–7,350	0.01
DeoR family protein/alkaline phosphatase (KP1_RS12850)	10.5^b^	1.62–67.9	0.014			
Deoxyribose-phosphate aldolase (KP1_RS12840)	5.79^b^	1.19–28.2	0.03			
Hypothetical protein (KPNJ1_01715)	5.08^b^	1.27–20.2	0.021	16.9^c^	1.59–179	0.019
Putative deoxygluconate dehydrogenase (KPN_01782)	4.5^b^	1.34–15	0.015	17.8^c^	2.2–143	0.007

a OR, odds ratio; CI, 95% confidence interval; *P*, *P* value.

b Bacterial gene odds ratios and *P* values after adjusting for above covariates.

c Odds ratio in combined model with five clinical covariates above and three bacterial genes.

### Pathogenicity-associated locus sequencing.

To identify bacterial genes that are associated with infection in this cohort, a reference sequence “pan-genome” was created. This was comprised of the entire genomes of five K. pneumoniae strains that are representative of pathogenic isolates with different virulence potentials and whose genome sequences are publicly available (23–25). MGH 78578 is a hospital-acquired strain of K. pneumoniae that was isolated from a patient with pneumonia. NTUH-K2044 is a hypervirulent strain that caused a liver abscess and meningitis (26). NJST258_1 is a Klebsiella pneumoniae carbapenemase (KPC)-producing sequence type 258 (ST258) strain from a urinary tract infection. KP342 is an isolate of Klebsiella variicola (9). KPPR1 is a genetically tractable strain frequently used in mouse models of infection (23). There were 3,910 orthologous genes identified as common to all five strains and considered the core genome for this analysis (see Fig. S1 and Data Set S1 in the supplemental material). The accessory genomes ranged from approximately 1,100 genes to just under 1,900 genes, and they represent an average of 26% of each genome.

10.1128/mSystems.00015-18.2 FIG S1  Pathogenicity-associated locus sequencing (PAL-Seq). (A) Genes from five reference genomes are strung together to create a “pan-genome”. Orthologous pan-genome genes are collapsed into gene loci. (B) Isolates are then sequenced using the Illumina HiSeq platform, and raw reads are mapped to the pan-genome, allowing us to determine the presence or absence of each gene in each genome. (C) Gene frequencies are then measured and compared between colonizing and disease-causing groups. Download FIG S1, TIF file, 0.6 MB. Copyright © 2018 Martin et al. 2018 Martin et al. This content is distributed under the terms of the Creative Commons Attribution 4.0 International license.

10.1128/mSystems.00015-18.10 DATA SET S1  Gene alignment counts and accession numbers. Download DATA SET S1, XLSX file, 13.5 MB. Copyright © 2018 Martin et al. 2018 Martin et al. This content is distributed under the terms of the Creative Commons Attribution 4.0 International license.

To assess the differences in gene frequencies between the case and control isolates, all 114 clinical isolates were sequenced, and read mapping to the pan-genome was performed. Strain KPPR1 was used as a positive control and had a mapping ratio of 98.9%. Reads from each sequenced sample were mapped to the pan-genome. Samples Kp499, Kp723, and Kp891 had the lowest mapping ratios, 66.7, 65.7, and 63%, respectively, suggesting that these strains are significantly different from the strains included in the pan-genome and are likely K. quasipneumoniae (Data Set S1). Sixteen samples had most of their reads mapped to strain KP342, indicating that they are K. variicola. The remaining 95 samples have most of their alignments equally distributed in strains KPPR1, MGH 78578, NJST258_1, and NTUH-K2044 (Data Set S1). To determine the lineage of each isolate, multilocus sequence typing (MLST) was performed and integrated with a reference set of MLST sequences from each species (Fig. S2A). This confirmed that the strain set contained 95 K. pneumoniae strains (34 infecting strains and 61 colonizing strains), 16 K. variicola strains (3 infecting strains and 13 colonizing strains), and 3 K. quasipneumoniae strains (1 infecting strain and 2 colonizing strains) (Fig. S2A). To assess the gene content of each strain’s accessory genome, normalized counts were summed for each gene and dichotomized as present or absent based on k-means analysis (Fig. S3). As validation of the k-mean analysis, PAL-seq successfully detected 5,016 of the 5,102 known KPPR1 genes and was negative for 3,157 of the 3,195 genes in the pan-genome that KPPR1 does not possess (sensitivity of 98.3%; specificity of 98.8%). Hierarchical clustering of present and absent genes in the accessory genomes (Fig. S4), as well as principal-component analysis (PCA) analysis of the accessory genomes (Fig. S5), also distinguished three groups based on species.

10.1128/mSystems.00015-18.3 FIG S2  Phylogenetic trees. (A) Clinical isolates analyzed using PAL-Seq were compared to reference isolates from each of the three identified Kp groups based on MLST sequence. The resulting phylogenetic tree shows three distinct branches, which distinguish Kp phylogroups: Kp phylogroup I, K. pneumoniae (green); Kp phylogroup II, K. quasipneumoniae (blue); Kp phylogroup III, K. variicola (red). Reference isolates are shown in darker color, while clinical PAL-Seq isolates are shown in lighter color. (B) Phylogenetic tree based on whole-genome variants. Whole-genome sequencing was used to assess variants among isolates, and a phylogenetic tree was generated, with the presence of tellurite resistance loci indicated in orange. The tellurite resistance loci are spread throughout all three species and do not appear to cluster in any one branch, indicating that its presence is not lineage associated. Download FIG S2, TIF file, 2 MB. Copyright © 2018 Martin et al. 2018 Martin et al. This content is distributed under the terms of the Creative Commons Attribution 4.0 International license.

10.1128/mSystems.00015-18.4 FIG S3  Binary classification of counts. A representative graph of normalized count sums versus density of gene bins with that count is shown for isolate Kp1711. A k-means clustering approach was applied to binarize the data to represent either “present” (a value of 1; shown in red) or “absent” (a value of 0; shown in blue) for each gene bin in that particular sequenced isolate. The inset pie chart shows the fractions of total gene bins present and absent in isolate Kp 1711. Download FIG S3, TIF file, 2.9 MB. Copyright © 2018 Martin et al. 2018 Martin et al. This content is distributed under the terms of the Creative Commons Attribution 4.0 International license.

10.1128/mSystems.00015-18.5 FIG S4  Hierarchical clustering of accessory genes. On the basis of the binary classification data, 3,169 bins were classified as present, and 703 bins were classified as absent, in all samples. After removal of these uniform bins, the remaining bins underwent hierarchical clustering of their classification, shown as a heatmap in which white and black represent absent and present, respectively. The column dendrogram can be viewed as a measurement of similarity between samples. The Kp phylogroup is indicated for each cluster. Download FIG S4, TIF file, 0.8 MB. Copyright © 2018 Martin et al. 2018 Martin et al. This content is distributed under the terms of the Creative Commons Attribution 4.0 International license.

10.1128/mSystems.00015-18.6 FIG S5  Principal-component analysis of normalized count sum data. Instead of binary classification, original normalized count sum data were used in PCA to see whether samples can be separated in clusters. The data were first processed by in-row quantile normalization, and PCA was performed between columns. Principal component 1 versus principal component 2 was plotted. The clustering of samples is consistent with what the alignment distribution reflects. Stool samples (blue), blood samples (red), and respiratory samples (green) are indicated. The KPPR1 lab strain is shown in black. Download FIG S5, TIF file, 0.4 MB. Copyright © 2018 Martin et al. 2018 Martin et al. This content is distributed under the terms of the Creative Commons Attribution 4.0 International license.

### Bacterial genes significantly associated with infection.

To determine whether there were significant differences in the frequency of any genes between case isolates and control isolates, a conditional logistic regression was performed using the binary classification. This analysis was limited to genes with frequencies of 5 to 95% within our sample set. After ranking by *P* value, certain genes clustered together based on their frequencies and sequential location in the reference sequence. Analysis of gene annotations and their locations in reference genomes indicated that these genes were likely located within operons. To facilitate further analysis, genes from the ten most significant operons (Table 3) were collapsed into one representative gene, with five loci as potential virulence factors and five as potential protective factors.

**TABLE 3:** Genes present in significantly different frequencies between case and control patients^a^

Reference genome gene locus tag	Annotation^b^	Frequency, no. (%)		*P* value
		Case^c^ (*n* = 38)	Control^d^ (*n* = 76)	
*KPK_RS16860*, *VK055_1478*, *KP1_RS09340*	*Iron ABC transporter permease*	*4 (10.5)*	*29 (38.1)*	*0.004369*
***KP1_RS12850*, *KPNJ1_RS13400*, *KPN_01704***	***DeoR family protein/alkaline phosphatase***	***32 (84.2)***	***43 (56.6)***	***0.005060***
*KPK_RS16855*, *VK055_1477*, *KP1_RS09345*	*Amino acid ABC transporter substrate-binding protein*	*4 (10.5)*	*28 (36.8)*	*0.005286*
*KPK_RS16810*, *VK055_1467*, *KP1_RS09395*^e^	*PTS fructose transporter subunit IIA*	*5 (13.1)*	*30 (39.4)*	*0.006209*
KPK_RS16815, VK055_1468, KP1_RS09390	PTS fructose transporter subunit IIC	5 (13.1)	30 (39.4)	0.006209
KPK_RS16825, VK055_1470, KP1_RS09380	Formate acetyltransferase	5 (13.1)	30 (39.4)	0.006209
KPK_RS16830, VK055_1471, KP1_RS09375	Pyruvate formate lyase II Activase	5 (13.1)	30 (39.4)	0.006209
KPK_RS16840, VK055_1473, KP1_RS09365	Phytanoyl-CoA dioxygenase	5 (13.1)	30 (39.4)	0.006209
KPK_RS16845, VK055_1476, KP1_RS09360	AraC family transcriptional regulator	5 (13.1)	30 (39.4)	0.006209
KPK_RS16850, VK055_1475, KP1_RS09355	Membrane protein	5 (13.1)	30 (39.4)	0.006209
***KPN_01782***	***Deoxygluconate dehydrogenase***	***26 (68.4)***	***30 (39.4)***	***0.007139***
*KPK_RS16865*, *VK055_1479*, *KP1_RS09335*	*Iron ABC transporter substrate-binding protein*	*5 (13.1)*	*30 (39.4)*	*0.007219*
*KPK_RS16820*, *VK055_1469*, *KP1_RS09385*^f^	*PTS fructose transporter subunit IIB*	*6 (15.8)*	*32 (42.1)*	*0.008326*
KPK_RS16835, VK055_1472, KP1_RS09370	PTS fructose transporter subunit IIB	6 (15.8)	32 (42.1)	0.008326
***KP1_RS12840*, *KPNJ1_RS13410*, *KPN_01702***^g^	***Deoxyribose-phosphate aldolase***	***31 (81.5)***	***43 (56.6)***	***0.009493***
KP1_RS12835, KPNJ1_RS13415, KPN_01701	Alkaline phosphatase/deoR	31 (81.5)	43 (56.6)	0.009493
KP1_RS12830 KPNJ1_RS13420, KPN_01700	Allulose-6-phosphate 3-epimerase	31 (81.5)	43 (56.6)	0.009493
KP1_RS12825, KPNJ1_RS13425, KPN_01699	Carbohydrate kinase (ribokinase)	31 (81.5)	43 (56.6)	0.009493
KP1_RS12820, KPNJ1_RS13430, KPN_01698	Permease	31 (81.5)	43 (56.6)	0.009493
***KP1_RS26720*, *KPK_RS27195***^h^	***Tellurium resistance protein TerF***	***10 (26.3)***	***6 (7.9)***	***0.012875***
KP1_RS26700, KPK_RS27215	Tellurium resistance protein TerB	10 (26.3)	6 (7.9)	0.012875
KP1_RS26655, KPK_RS27260	Tellurium resistance protein TerW	10 (26.3)	6 (7.9)	0.012875
KP1_RS26650, KPK_RS27270	Tellurium resistance protein TerY	10 (26.3)	6 (7.9)	0.012875
***KPNJ1_01715***	***Hypothetical protein***	***13 (34.2)***	***10 (13.2)***	***0.013956***

a Genes in operons were collapsed into one representative gene. Representative potential protective factors are shown in italic type, and potential risk factors are shown in italic boldface type.

b PTS, phosphotransferase system; CoA, coenzyme A.

c Patients with bloodstream infection or pneumonia.

d Patients with asymptomatic rectal colonization.

e Representative gene for KPK_RS16810, -16815, -16825, -16830, -16840, -16845, and -16850.

f Representative gene for KPK_RS16820 and -16835.

g Representative gene for KP1_RS12820 to -12840.

h Representative gene for KP1_RS26720, -26700, -26655, and -26650.

To determine which loci were significantly and independently associated with infection in our cohort of patients, each representative gene was individually adjusted for the six patient-level variables from the clinical model. The five potential pathogenicity-associated loci (PALs) were significantly associated with K. pneumoniae infection (Table 2), independent of patient-level variables that differed between cases and controls. TerF (KP1_RS26720) is representative of the *ter* tellurite resistance locus. The DeoR family regulatory protein (KP1_RS12850) and deoxyribose-phosphate aldolase (KP1_RS12850) had slightly different gene frequencies but are both part of the same sugar utilization locus (Fig. S6). A hypothetical protein (KPNJ1_01715) and a putative deoxygluconate dehydrogenase (KPN_01782) that may act as an oxidoreductase were also associated with infection. In contrast, the five loci that were potentially protective on unadjusted analysis (Table 2) were not associated with whether or not a patient was infected or asymptomatically colonized after adjustment for patient-level variables.

10.1128/mSystems.00015-18.7 FIG S6  Pathogenicity-associated loci (PALs). Two gene operons identified by PAL-Seq are shown, the putative sugar utilization locus and the tellurite resistance locus. Genes not identified in PAL-Seq are shown in gray. Genes deleted for characterization are shown in boldface type. Download FIG S6, TIF file, 0.4 MB. Copyright © 2018 Martin et al. 2018 Martin et al. This content is distributed under the terms of the Creative Commons Attribution 4.0 International license.

### Adjusting bacterial genes for host factors.

To determine whether the five PALs identified by PAL-Seq would remain significantly associated with infection after adjusting for differences between cases and controls (Table 2 and Fig. 1A), representative genes of the five PALs were added to the clinical model built above followed by backwards elimination. This process retained three genes and removed central line in the final, combined model (Table 2). As mentioned above, another reason that central line was not retained in this final model is that it only slightly improved the performance of the initial model with a borderline significance (*P* = 0.054 for likelihood ratio test). This model identified the representative genes for tellurite resistance, deoxygluconate dehydrogenase, and the hypothetical protein as highly associated with disease when adjusted for patient variables from the previous model (Table 2). This revised model also fit better with the data set than the model based only on patient factors (AUROC of 0.95 versus 0.88; *P* = 0.011) (Fig. 1B). In this model, the tellurite resistance locus had the greatest association with infection (odds ratio [OR], 157; 95% confidence interval [95% CI], 3.34 to 7,350; *P* = 0.01) followed by the presence of a fluid and electrolyte disorder (OR, 22.9; 95% CI, 1.6 to 329; *P* = 0.021), deoxygluconate dehydrogenase (OR, 17.8; 95% CI, 2.2 to 143; *P* = 0.007), and the hypothetical protein gene (OR, 16.9; 95% CI, 1.59 to 179; *P* = 0.019).

### Phylogenetic and phenotypic characterization of the tellurite resistance locus.

**FIG 2:**
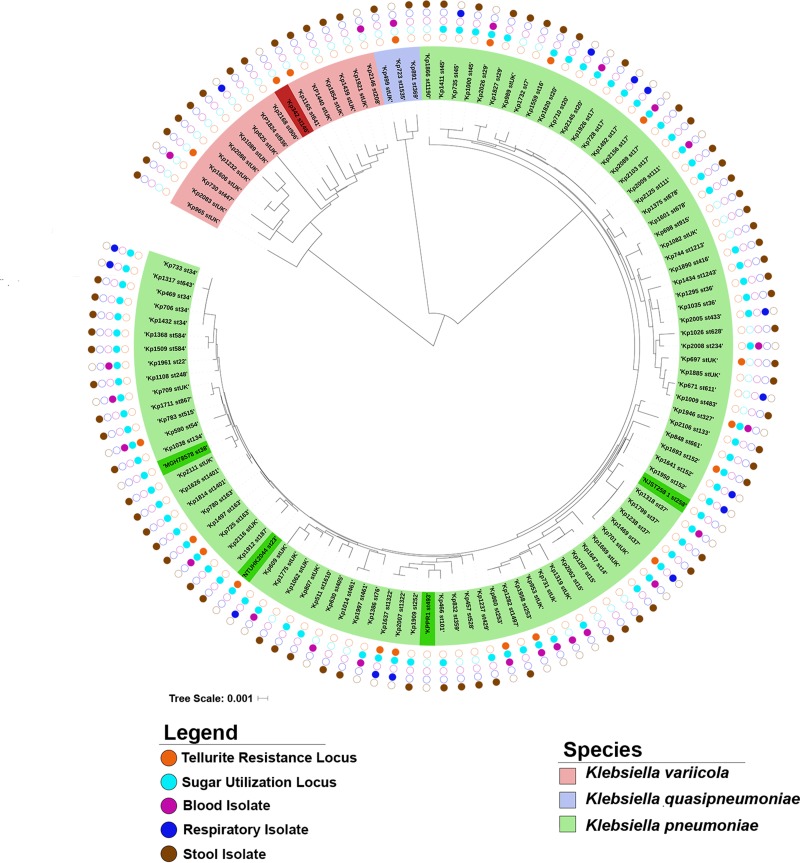
Phylogenetic analysis of clinical isolates. Multilocus sequence typing (MLST) was performed on all clinical isolates plus all five reference strains. The locus type, isolate type, and *Klebsiella* species are indicated by the colors shown in the keys. Reference isolates are shown in dark green or dark red. The bar shows 0.001 nucleotide substitution per position.

The *ter* tellurite resistance locus has been associated with specific hypervirulent sequence types (STs), suggesting that it may be a marker of lineage and not an independent predictor of clinical K. pneumoniae infection (27). To determine whether the *ter* locus is associated with certain lineages in this cohort, multilocus sequence typing was performed. A phylogenetic tree based on MLST demonstrated that the *ter* locus is present across multiple STs and does not appear to cluster among any one ST or any closely related group of STs (Fig. 2). A phylogenetic tree based on sequence variants identified by whole-genome sequencing (WGS), although not identical to that of MLST, confirmed that possession of the *ter* locus is not lineage associated (Fig. S2B). In fact, this locus was found across K. pneumoniae, K. quasipneumoniae, and K. variicola isolates.

**FIG 3:**
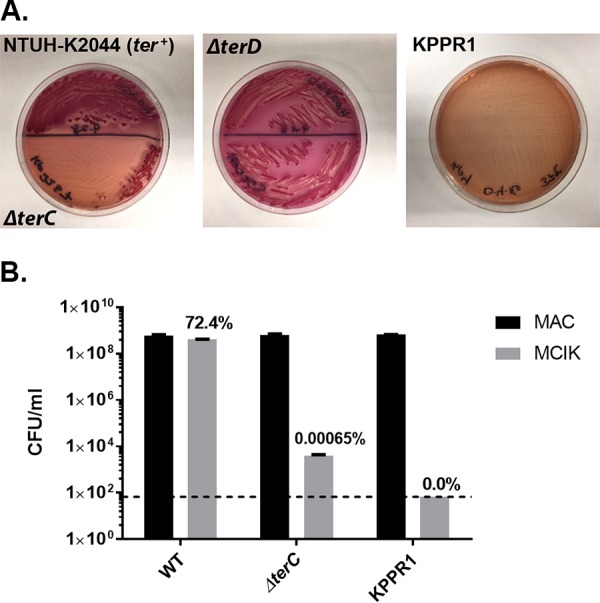
Deletion of *terC* confers a tellurite-sensitive phenotype. (A) K. pneumoniae NTUH-K2044 (WT), Δ*terC* mutant, Δ*terD* mutant, and KPPR1 (no *ter* locus) were plated on MacConkey-inositol-potassium tellurite (MCIK) agar (3 µg potassium tellurite/ml) and visualized for inhibition of growth. (B) Quantification of growth on MCIK agar compared to growth on MacConkey (MAC) agar was assessed. Data are values for two replicates. Error bars indicate standard error of the mean (SEM). Dashed line indicates the limit of detection for this assay.

To characterize the role of the *ter* locus in virulence, targeted mutagenesis of *terC* and *terD* was performed, which leads to an elimination of the tellurite-resistant phenotype in E. coli (28). These two genes were deleted individually on the pK2044 plasmid in the hypervirulent K. pneumoniae strain NTUH-K2044 that was part of the reference sequence for PAL-Seq and has been previously shown to cause pneumonia in an animal model (29). To determine whether deletion of either gene leads to a tellurite-sensitive phenotype, growth of mutants on MacConkey-inositol-potassium tellurite (MCIK) agar was assessed (30). Deletion of *terC* but not *terD* led to a tellurite-sensitive phenotype as determined by growth on MCIK agar (Fig. 3A), with 0.00065% recovery compared to MacConkey (MAC) agar (Fig. 3B). To determine whether removal of *terC* led to an *in vivo* fitness defect, mice were infected with a 1:1 ratio of mutant to wild-type (WT) bacteria using a pneumonia model of infection (see Materials and Methods), and a competitive index was calculated. No significant fitness defect was seen in the lungs at 24 h (Fig. S7A) or 48 h (Fig. S7B) postinfection.

10.1128/mSystems.00015-18.8 FIG S7  Competitive index of the *terC* mutant compared to the WT. A cochallenge infection was performed on C57BL/6 mice to determine whether there is an *in vivo* fitness defect in the Δ*terC* strain, and the competitive index was calculated at 24 h (A) (*n* = 16) and 48 h (B) (*n* = 11) postinfection. The mean of the log-transformed competitive indices was compared to a hypothetical value of 0 using a one-sample *t* test for each group. Download FIG S7, TIF file, 0.2 MB. Copyright © 2018 Martin et al. 2018 Martin et al. This content is distributed under the terms of the Creative Commons Attribution 4.0 International license.

### Identification of the sugar utilization locus substrate.

**FIG 4:**
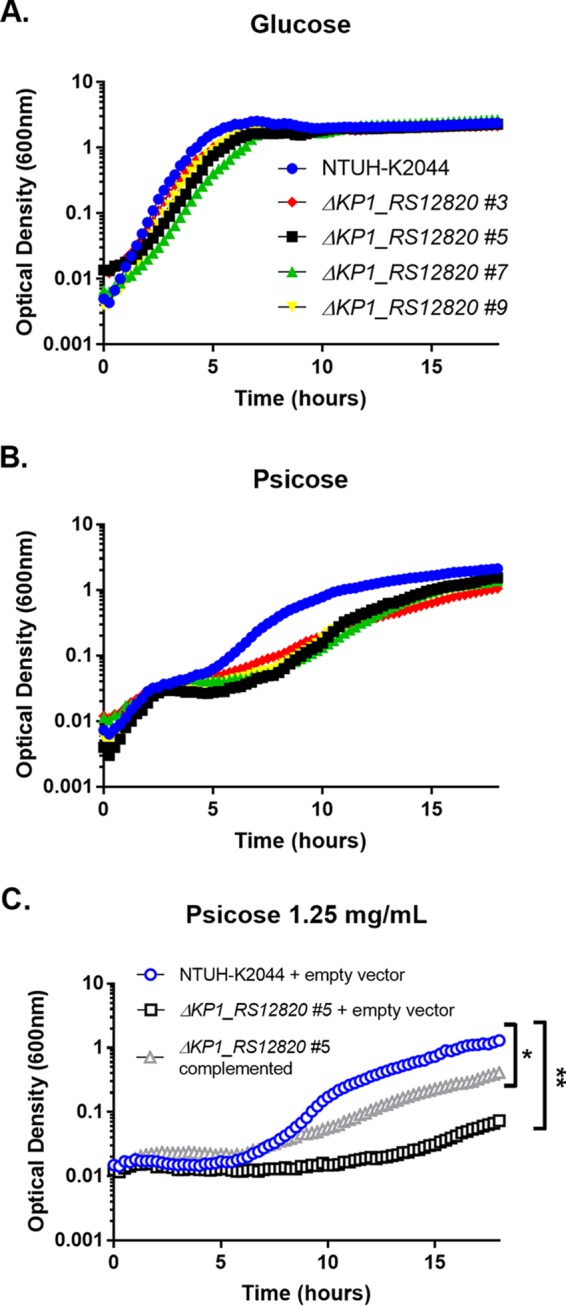
Deletion of *KP1_RS12820* affects growth in psicose. (A and B) Each well contained either glucose at a final concentration of 5 mg/ml (A) or psicose at a final concentration of 5 mg/ml (B). (C) WT and empty vector, *ΔKP1_RS12820* clone 5 (#5) and empty vector, or the complemented mutant were also tested in psicose (1.25 mg/ml). Diluted inoculum was added to each well 1:1. Growth was recorded using optical density at 600 nm over 18 h. Data points shown are the means for three technical replicates and are representative of two or more experiments. Statistics were calculated using one-way ANOVA for multiple comparisons. Values that are significantly different are indicated by brackets and asterisks as follows: *, *P* = 0.0335; **, *P* = 0.0079.

To characterize the function of the sugar utilization locus associated with infection and represented by genes *KP1_RS12840* and *KP1_RS12850* (Table 2), a deletion mutation of the putative sugar permease gene *KP1_RS12820* was constructed. This gene had the same gene frequency in patients as *KP1_RS12840*, and permeases mediate uptake of sugars into bacteria. To identify the sugar substrate of this locus, the WT and Δ*KP1_RS12820* mutant (Kp2241; clone 3) were grown in minimal medium with and without the addition of various sugars. Growth was equivalent on glucose (Fig. 4A), but the most prominent difference in growth patterns between the WT and Δ*KP1_RS12820* mutant was seen on psicose (Fig. 4B). Whole-genome sequence variant analysis of Kp2241 identified a deletion mutation in *cyoD* (NC_012731.1, position 1216091), but three additional mutant clones had comparable growth to WT when grown in M9 minimal medium with glucose (Fig. 4A). All mutant clones had a growth defect on psicose compared to the WT (Fig. 4B). Complementation using a plasmid encoding *KP1_RS12820* of a mutant (Kp4174; clone 5) with no detectable secondary mutations resulted in partial rescue of the growth defect (Fig. 4C). These results indicate that *KP1_RS12820*, *KP1_RS12840*, and *KP1_RS12850* are part of a psicose utilization locus.

### Contribution of the psicose utilization locus to fitness in a murine pneumonia model.

**FIG 5:**
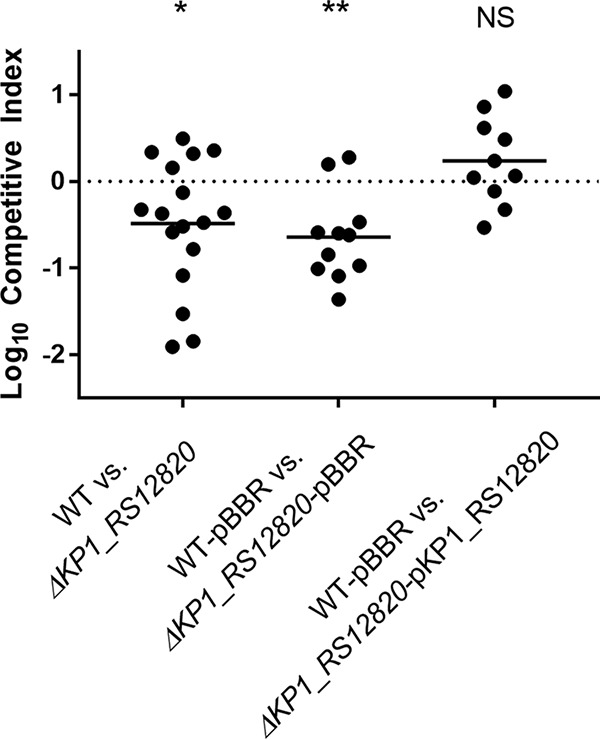
Deletion of putative sugar permease *KP1_RS12820* leads to an *in vivo* fitness defect. C57BL/6 mice were inoculated intrapharyngeally with a 1:1 ratio of NTUH-K2044 (WT) and *ΔKP1_RS12820* bacteria (1 × 10^4^ CFU/mouse total) or with the WT bacteria carrying the pBBR1MCS-5 plasmid in combination with *ΔKP1_RS12820* bacteria carrying pBBR1MCS-5 or pBBR1MCS-5 with the *ΔKP1_RS12820* gene. A competitive index was calculated based on the ratios of WT and mutant input CFU and output CFU. The mean values (indicated by the short black lines) of the log-transformed competitive indices (10 to 17 mice in each group; two or three experiments) were compared to a hypothetical value of zero using a one-sample *t* test for each group. NS, not statistically significant.

To determine whether deletion of *KP1_RS12820* leads to an *in vivo* fitness defect, mice were infected with a 1:1 ratio of mutant to wild-type bacteria using a pneumonia model of infection used previously for competitive infections (31). After 24 h, bacterial density of each strain in the lung was assessed and a competitive index was calculated. The Δ*KP1_RS12820* mutant Kp4174 had a significant *in vivo* fitness defect in a pneumonia model compared to the wild type, and complementation restored fitness of the mutant (Fig. 5). The sugar transport locus was present in 77.3% (75/97) of K. pneumoniae clinical isolates in our cohort, but not in K. quasipneumoniae or K. variicola (Fig. 2). Despite its high prevalence in K. pneumoniae, multivariable analysis to control for bacterial species showed that the presence of the psicose locus remains associated with infection independent of species (Table S1).

10.1128/mSystems.00015-18.9 TABLE S1  Multivariable model for psicose utilization locus association with infection. Download TABLE S1, DOCX file, 0.03 MB. Copyright © 2018 Martin et al. 2018 Martin et al. This content is distributed under the terms of the Creative Commons Attribution 4.0 International license.

## DISCUSSION

The objective of this study was to identify genes in the K. pneumoniae accessory genome that are associated with clinical infection after controlling for potentially confounding patient variables. Combining a case-control study design and comparative genomics method (PAL-Seq), five genetic loci were identified as significantly and independently associated with infection in the sample set. Six patient factors were also identified that were considered important for potential inclusion in the final model. The final model combining five of these patient factors and three K. pneumoniae genes had good fit with this sample set. The presence of the tellurite locus had the strongest association with infection, after adjustment for patient factors. A sugar utilization locus was also independently associated with human infection after adjustment for patient variables. Although it was not selected for inclusion in the final model, it improved the fitness of K. pneumoniae in a murine model of pneumonia. Our results suggest that evaluation of genes in the accessory genome of K. pneumoniae can identify those associated with HAIs, yielding novel insights into mechanisms of pathogenesis and providing potential diagnostic targets to predict infections.

The purpose of the initial clinical model was to enable identification of bacterial genes independently associated with infection by normalizing for any differences in patient factors between cases and controls. This normalization was important, as controls were drawn from the intensive care units (ICUs) and hematology/oncology wards, where rectal swab cultures were collected as part of routine infection prevention practices, whereas cases were drawn from additional wards in the hospital. The initial model identified white race as the only patient factor significantly different between groups, and it appears to be protective. This could be due to a difference in host genetics or an indirect marker of socioeconomic status, but testing those hypotheses is beyond the scope of our current study, which sought only to adjust for differences at the patient level in order to bolster our findings regarding K. pneumoniae genetics and infection. Our final multivariable model identified fluid and electrolyte disorders as a risk factor, consistent with findings in a previous study (15), but other factors differed. The differences in associated patient factors between these two studies may be due to the smaller sample size of the current study or the increased heterogeneity of the patient population. However, the adjusted model enabled detection of bacterial genes significantly associated with infection in this collection of patients, despite some cases and controls being drawn from different sources.

The plasmid-encoded tellurite resistance (*ter*) locus had the strongest association with infection by our PAL-Seq analysis. The antibacterial properties of tellurite have long been known (32). In K. pneumoniae and some other species, this resistance locus consists of two operons (*terZABCDEF* and *terWXY*) separated by seven uncharacterized putative open reading frames. The *ter* operon has been associated with hypervirulent K. pneumoniae clonal groups (27), but it appears to be associated with infection independent of K. pneumoniae lineage in our group of patients (Fig. 2 and see Fig. S2B in the supplemental material). The mechanism of bacterial tellurite resistance is unclear, although it may be linked to resistance to superoxide and other reactive oxygen species. Although tellurite is unlikely to be found in the human body, a tellurite resistance locus in Bacillus anthracis contributes to fitness in a bacteremia model (33). Although not required for a hypervirulent K. pneumoniae strain to cause murine pneumonia, it may contribute to pathogenesis at other sites of infections. Alternatively, it may be a robust genetic marker that is strongly linked to virulence-associated plasmids that vary in their combinations of virulence genes. Regardless, the corresponding tellurite resistance phenotype can be easily screened for in patient samples using either preformulated media or through simple modifications of MacConkey agar routinely used in clinical laboratories (27, 30).

Of the five representative genes independently associated with infection, both *KP1_RS12850* and *KP1_RS12840* appear to be part of the same putative sugar utilization locus. Their slightly different frequencies may be an artifact of the PAL-Seq method. Deletion of the permease gene in this locus impaired growth on d-psicose, indicating that this is the substrate (Fig. 4). This is consistent with a recent study that associated the same sugar utilization locus with psicose uptake and degradation in K. pneumoniae strains using comparative genomics (34). The permease mutant also had an *in vivo* fitness defect, providing experimental validation of this gene as a fitness factor (Fig. 5). d-Psicose is also known as allulose and is a C-3 epimer of d-fructose. d-Psicose/allulose is a rare sugar, occurring only in small quantities in nature. It is, however, encountered as a natural sweetener for food and drink (35). Upon consumption, d-psicose is absorbed in the small intestine and excreted in the urine (36), providing two potential sites where colonizing K. pneumoniae could encounter this substrate. Though K. pneumoniae is a frequent colonizer of the large intestine in humans, it has been commonly identified in cases of small intestine bacterial overgrowth (SIBO) (37, 38). It is possible that the presence of d-psicose could enhance intestinal K. pneumoniae colonization, although this has not been investigated. If true, this could identify d-psicose ingestion as a risk factor for progression to disease. However, the source and function of psicose during pneumonia are unclear.

A putative deoxygluconate dehydrogenase (*KPN_RS09590*) and a hypothetical protein (*KPNJ1_01715*) were also highly predictive of infection in the final multivariable model. Deoxygluconate dehydrogenases are enzymes categorized under EC 1.1.1.125 and are a class of oxidoreductases that are thought to play a role in pentose and glucuronate interconversions (39). Among the strains in our reference pan-genome, this gene was present only in strain MGH 78578, which caused pneumonia. It was present in 56 (49.1%) of our patient isolates (30 colonizing [39.4%] versus 26 infecting [68.4%]). The hypothetical protein nucleotide sequence *KPNJ1_01715* is less than 100 bp and was annotated only in NJST258_1 (a Klebsiella pneumoniae carbapenemase producer) in the reference pan-genome. At the time of this publication, *KPNJ1_01715* had been removed as an open reading frame from GenBank. This nucleotide sequence sits between two divergently transcribed genes, phosphoporin PhoE (*KPNJ1_RS08390* and *KP1_RS18000*) and phosphotransferase RcsD (*KPNJ1_RS08380* and *KP1_RS18005*), suggesting that it may be a regulatory region of these genes.

Although this study successfully identified genes independently associated with K. pneumoniae infection, there were some limitations to the PAL-Seq approach. The primary limitation is use of only five strains to make the pan-genome. Whole-genome sequencing indicates that the K. pneumoniae pan-genome (conserved and accessory genes) is open, indicating that new genes will continue to be identified (13). Therefore, it is probable that this analysis did not cover the breadth of genes represented among clinical isolates. Of the approximately 30,000 protein-coding sequences identified thus far, this study sampled just over 8,000. Similarly, this study did not include a K. quasipneumoniae strain in the pan-genome, though there were three patient isolates identified as K. quasipneumoniae, and PAL-Seq analysis showed that reads from these isolates mapped poorly to the pan-genome. Whole-genome sequencing of all three species has identified a 3 to 4% nucleotide divergence (across core genes) between phylogroups, compared to ~0.5% divergence within phylogroups, indicating that allelic differences may account for poor mapping of our K. quasipneumoniae isolates (13). It is possible that this affected the gene frequency calculations, though unlikely that it greatly affected the overall outcome, since only three isolates were identified as K. quasipneumoniae species. However, well-characterized, genetically tractable strains were chosen for the concatenated reference sequence that could be used to directly test fitness of mutants in an animal model. This approach facilitated translation from association with human infection to phenotypic characterization *in vitro* and in animal models of infection.

By combining clinical modeling with bacterial comparative genomics, this study identified several K. pneumoniae loci that are independently and significantly associated with infection in hospitalized patients. This bacterial GWAS provides a proof of principle in using clinical isolates to gain insight into bacterial pathogenesis. Along with bacterial genes, host differences are potentially another strong determining factor in whether a patient goes on to develop infection. To apply these findings in the clinical microbiology laboratory, the associations must be validated in a larger patient cohort, and the contributions of host factors need to be identified and accounted for in interpretation of bacterial genetic results. Then, diagnostic assays could be designed and validated to screen for at-risk patients who could benefit from infection prevention interventions.

## MATERIALS AND METHODS

### Study design.

The objective of this study was to identify Klebsiella pneumoniae genes associated with clinical infection compared to asymptomatic colonization. The study was conducted at University Hospital, a tertiary care hospital in Ann Arbor, Michigan, with more than 1,000 beds and part of Michigan Medicine. Approval for this study was granted by the Institutional Review Board of the University of Michigan Medical School. Patient demographic characteristics and clinical information were obtained through the electronic medical record (EMR). The research subjects were from two overlapping groups: patients (aged ≥16 years) in the intensive care unit (ICU) or hematology/oncology wards who had surveillance rectal swabs collected for infection prevention purposes and in-patients from any ward with a clinical culture positive for K. pneumoniae from 30 July 2014 to 31 October 2014.

A case-control study was performed with 1:2 matching. Infected patients (cases) with bacteremia were identified based on positive blood cultures, and cases of pneumonia were identified based on a positive K. pneumoniae respiratory culture and meeting Infectious Diseases Society of America (IDSA) diagnostic criteria (22). Tracheal aspirates that met the IDSA criteria were included. No tracheal aspirates contained multiple pathogens. Asymptomatically colonized patients (controls) were identified based on rectal colonization with K. pneumoniae and no positive K. pneumoniae extraintestinal cultures within 90 days postcolonization. Controls were matched with cases based on sex, age range (within 10 years), and sample collection date range (within 3 weeks). Control pools were generated using SAS 9.4 (SAS Institute, Cary, NC), and final matches were randomly selected using Microsoft Excel.

### Bacterial strains and media.

Klebsiella pneumoniae NTUH-K2044 was provided by Jin-Town Wang at the National Taiwan University College of Medicine. Strain NTUH-K2044 and derived mutants were cultured at 30°C on LB agar supplemented with kanamycin (25 µg/ml), spectinomycin (50 µg/ml), or gentamicin (6 to 10 µg/ml) as indicated. Isolates were also cultured in LB broth at 37°C with shaking. M9 minimal medium (2×) was made by adding MgSO_4_ (final concentration of 2 mM) and CaCl_2_ (final concentration of 0.1 mM) to M9 minimal salts (Life Technologies, Carlsbad, CA).

### Construction of mutants.

Lambda Red mutagenesis was performed as previously described (31, 40) with the following modifications. Electrocompetent cells were prepared by culture in LB broth containing a final concentration of 0.5 µM EDTA at 37°C with shaking until an optical density at 600 nm (OD_600_) between 0.5 and 0.6 was attained. The culture was placed on ice for 45 min and centrifuged in sterile cold bottles at 8,000 rpm for 15 min at 4°C. The supernatant was decanted, and bacteria were washed and centrifuged in ice-cold sterile volumes of 25 ml of 1 mM HEPES, 25 ml of distilled water, and 10 ml of 10% glycerol. Pellets were brought to a final density of 2 × 10^10^ CFU/ml and stored in 50-µl aliquots at −80°C. A modified pKD46 plasmid carrying a gene encoding spectinomycin resistance was electroporated into strain NTUH-K2044 using a 0.1-cm-gap cuvette at 1.8 kV, 400 Ω, and 25 µF, with a Bio-Rad Micropulser. Cells were recovered in room temperature SOC medium (S1797, Sigma-Aldrich, St. Louis, MO) and incubated overnight at 30°C with shaking. Electrocompetent cells containing the pKD46 plasmid were prepared as described above, except cultures were grown for approximately 4 h at 30°C in LB broth containing spectinomycin, 50 mM l-arabinose, and 0.5 µM EDTA.

### Complementation of mutants.

To complement the *KP1_RS12820* mutants, PCR products containing the open reading frame were inserted into pCR 2.1 using TOPO TA cloning (Life Technologies, Carlsbad, CA) and subsequently ligated into pBBR1MCS-5 following digestion with HindIII and Xho1. The complementation plasmid was electroporated into wild-type (WT) and mutant K. pneumoniae.

### Murine pneumonia model.

Six- to 11-week-old mice C57BL/6 mice were anesthetized with isoflurane and then inoculated intrapharyngeally with ~1 × 10^4^ CFU of bacteria per mouse. The mice were inoculated with a 1:1 mixture of NTUH-K2044 (WT) and mutant bacteria, either Δ*KP1_RS12820* or Δ*terC* mutant, WT with empty vector and mutant with empty vector (pBBR1MCS-5), or WT with empty vector and the complemented mutant. After 24 or 48 h, mice were euthanized by CO_2_ asphyxiation and lungs were removed, homogenized in 1 ml PBS, and cultured on selective agar. The competitive index was calculated as follows: (mutant lung CFU/WT lung CFU)/(mutant inoculum CFU/WT inoculum CFU). All care and use of animals were in accordance with institutional guidelines.

### Growth curves.

Bacterial strains were cultured overnight in LB broth. Culture concentrations were normalized, washed in 2× minimal medium, and diluted to a final concentration of 1.4 × 10^7^ CFU/ml. Each well contained glucose or psicose at a final concentration of 5 mg/ml or 1.25 mg/ml. Diluted inoculum was added to each well 1:1 (1× final minimal medium concentration). Strains were cultured for 24 h at 37°C. Absorbance readings at 600 nm were taken every 15 min using an Eon microplate spectrophotometer with Gen5 software (BioTek, Winooski, VT).

### Bioscreen assay.

Bacterial strains were cultured overnight in LB broth either aerobically or in a vinyl anaerobic chamber (Coy Laboratory Products, Grass Lake, MI). On the following day, cultures were incubated in M9 (Life Technologies) medium with or without various carbon supplementation as described elsewhere (41, 42). Strains were cultured for 24 to 196 h at 37°C either aerobically or in the anaerobic chamber, respectively. Absorbance readings at 600 nm were taken every 15 min using an Eon microplate spectrophotometer with Gen5 software (BioTek, Winooski, VT).

### Statistical analysis.

Conditional logistic regression models were used to identify genes that have significantly different presence rates in cases and controls. This model takes into account the matched nature of the data, and a *P* value for each gene was obtained to indicate the significance of that gene.

The clinical and multivariable modeling was conducted in R version 3.3.1 (R Foundation for Statistical Computing, Vienna, Austria). Unless otherwise specified, a significance threshold of *P* < 0.05 was used for all analyses. Initial analyses included descriptive statistics and exploring various variable constructions for continuous variables and categorical variables with more than two levels. Bivariable analyses for the outcome of invasive infection (cases) were conducted using conditional logistic regression via the *survival* package, version 2.38 (43). Where different possible variable constructions existed, the ones with the best fit by *P* value on bivariable analyses were carried forward. Variables with *P* < 0.2 on these initial bivariable analyses were eligible for inclusion in a final multivariable model. The final clinical model was constructed through backwards elimination with a cutoff α of 0.05 for the likelihood ratio test. Interactions among variables in the final model were assessed and included if significant. Candidate genes from the pathogenicity-associated locus sequencing (PAL-Seq) analysis that associated with invasive infection were then adjusted individually for the variables in the clinical model. The candidate genes that remained significant after this initial adjustment were all added to the clinical model, and backwards elimination was again conducted to arrive at the final model that incorporated both patient-level and bacterial genetic features. Interaction testing proceeded as before. The overall fit of the multivariable models was assessed through construction of receiver operating characteristic curves (ROCs) and calculation of the area under the curve. Bootstrapped confidence intervals for specificity at each level of sensitivity were calculated and plotted for the ROCs via the package *pROC* version 1.8 (44). Multivariable model receiver operating characteristic curve (AUROCs) were compared using the DeLong method (45). Bacterial growth was compared by one-way analysis of variance (ANOVA). Competitive infections were evaluated for significance by a one-sample *t* test comparing the mean of the log-transformed competitive index to a theoretical value of 0.

### Cultivation and whole-genome sequence analysis of clinical isolates.

For details on cultivation and whole-genome sequence analysis of clinical isolates, see Text S1 in the supplemental material.

10.1128/mSystems.00015-18.1 TEXT S1  Supplemental Materials and Methods. Download TEXT S1, DOCX file, 0.04 MB. Copyright © 2018 Martin et al. 2018 Martin et al. This content is distributed under the terms of the Creative Commons Attribution 4.0 International license.

### Data availability.

Illumina sequence reads from bacterial whole-genome sequence have been deposited in the Sequence Read Archive (SRA, NCBI) under the accession numbers listed in Data Set S1 in the supplemental material.
