# Supplementary figures and images for: Identification of Pathogenicity-Associated Loci in Klebsiella pneumoniae from Hospitalized Patients
Source: mSystems. 2018 Jun 26;3(3):e00015-18. doi: 10.1128/mSystems.00015-18 (PMC6020474; doi:10.1128/mSystems.00015-18)

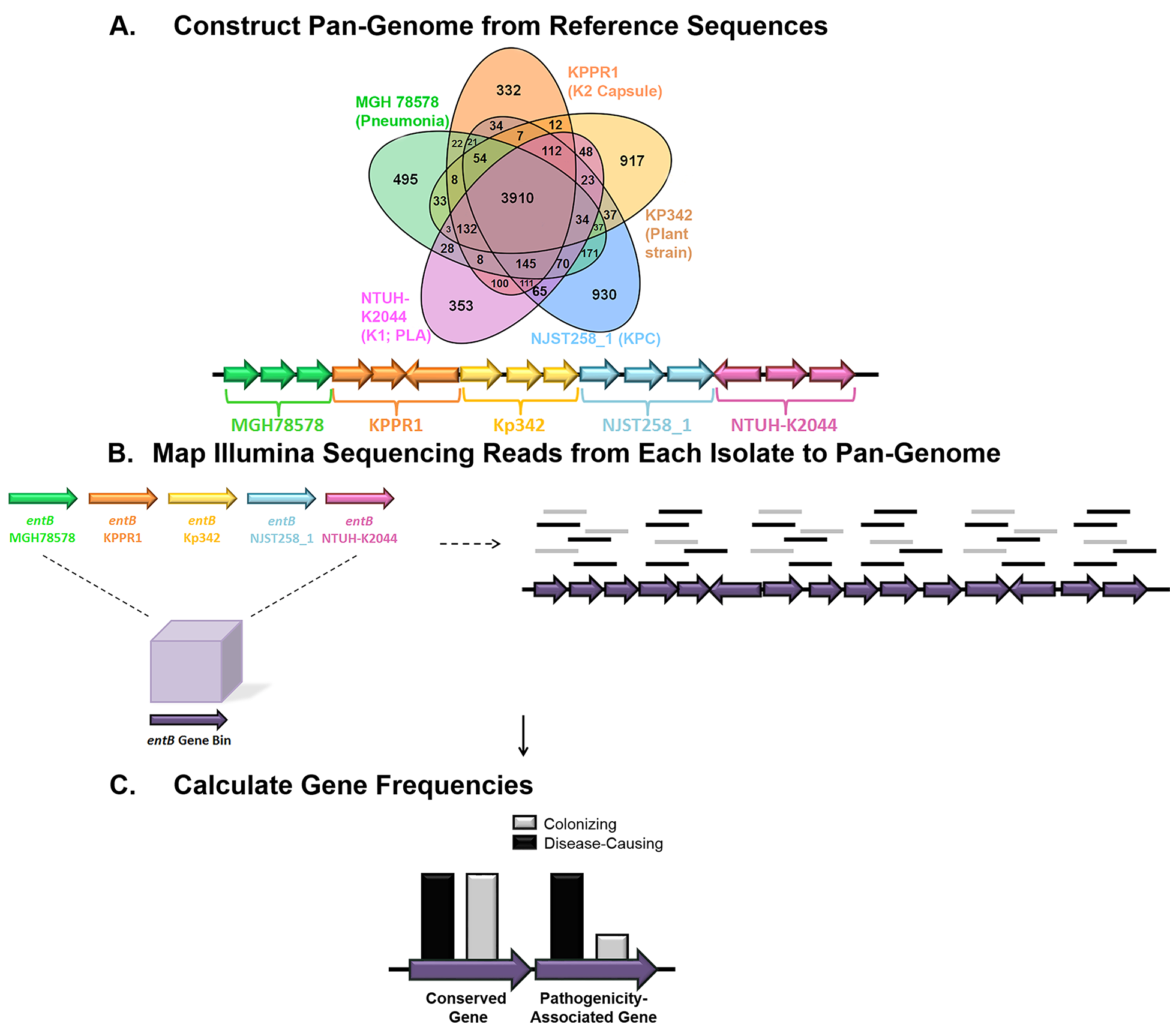

Supplement: FIG S1 [file sys003182238sf1.tif]

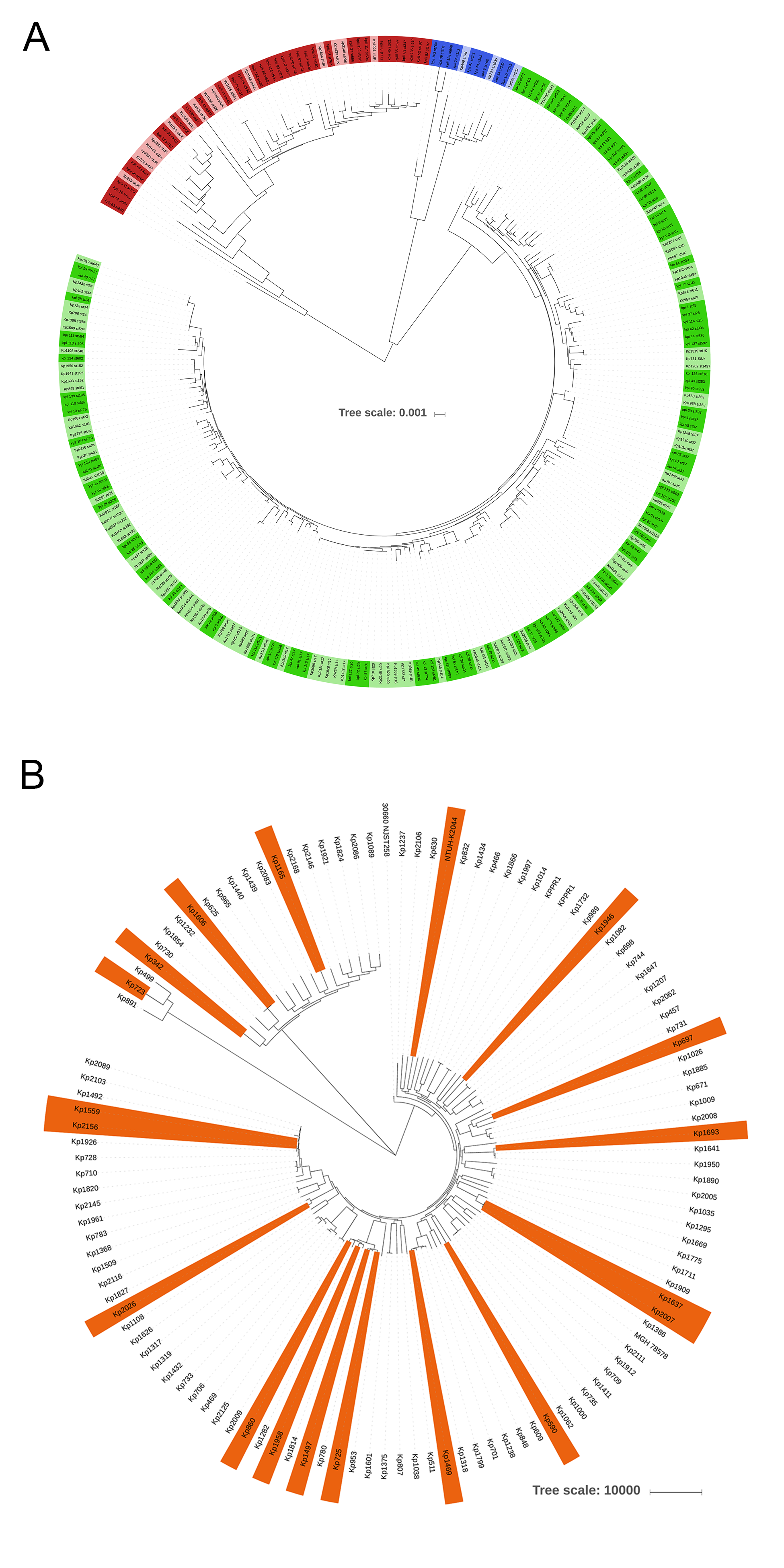

Supplement: FIG S2 [file sys003182238sf2.tif]

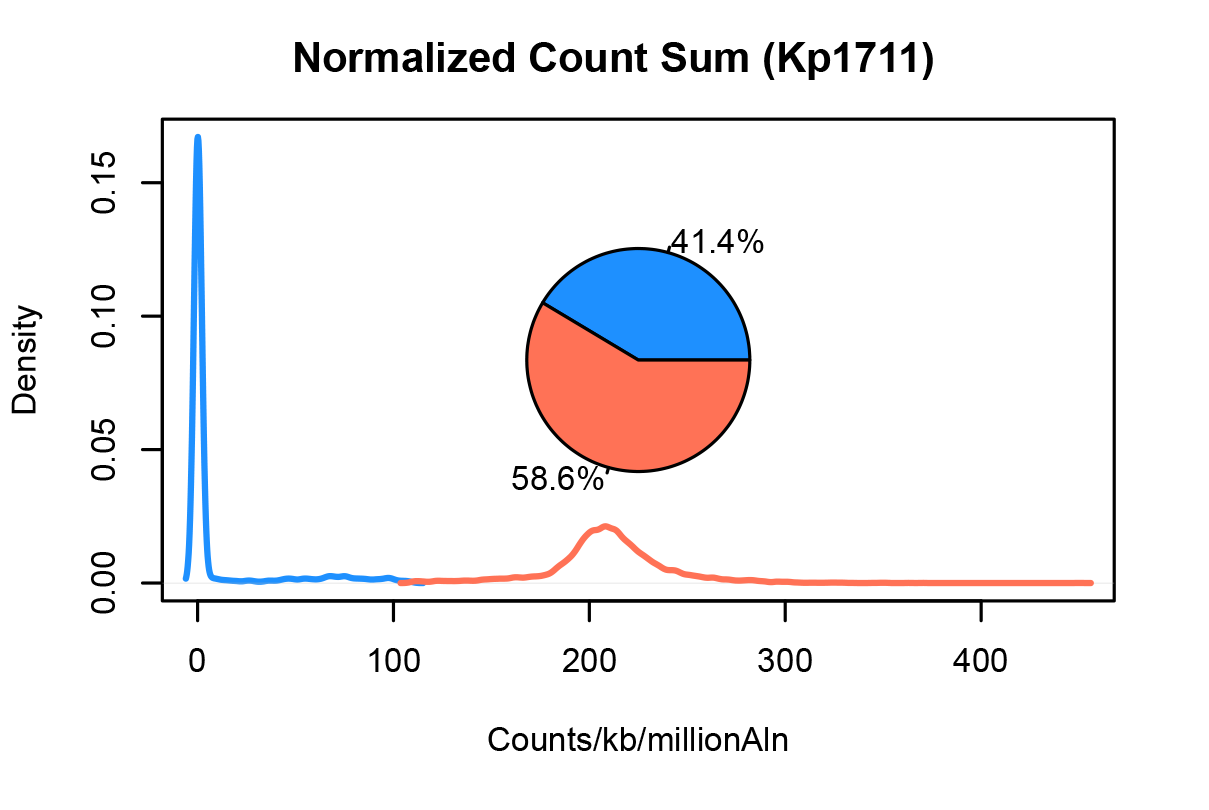

Supplement: FIG S3 [file sys003182238sf3.tif]

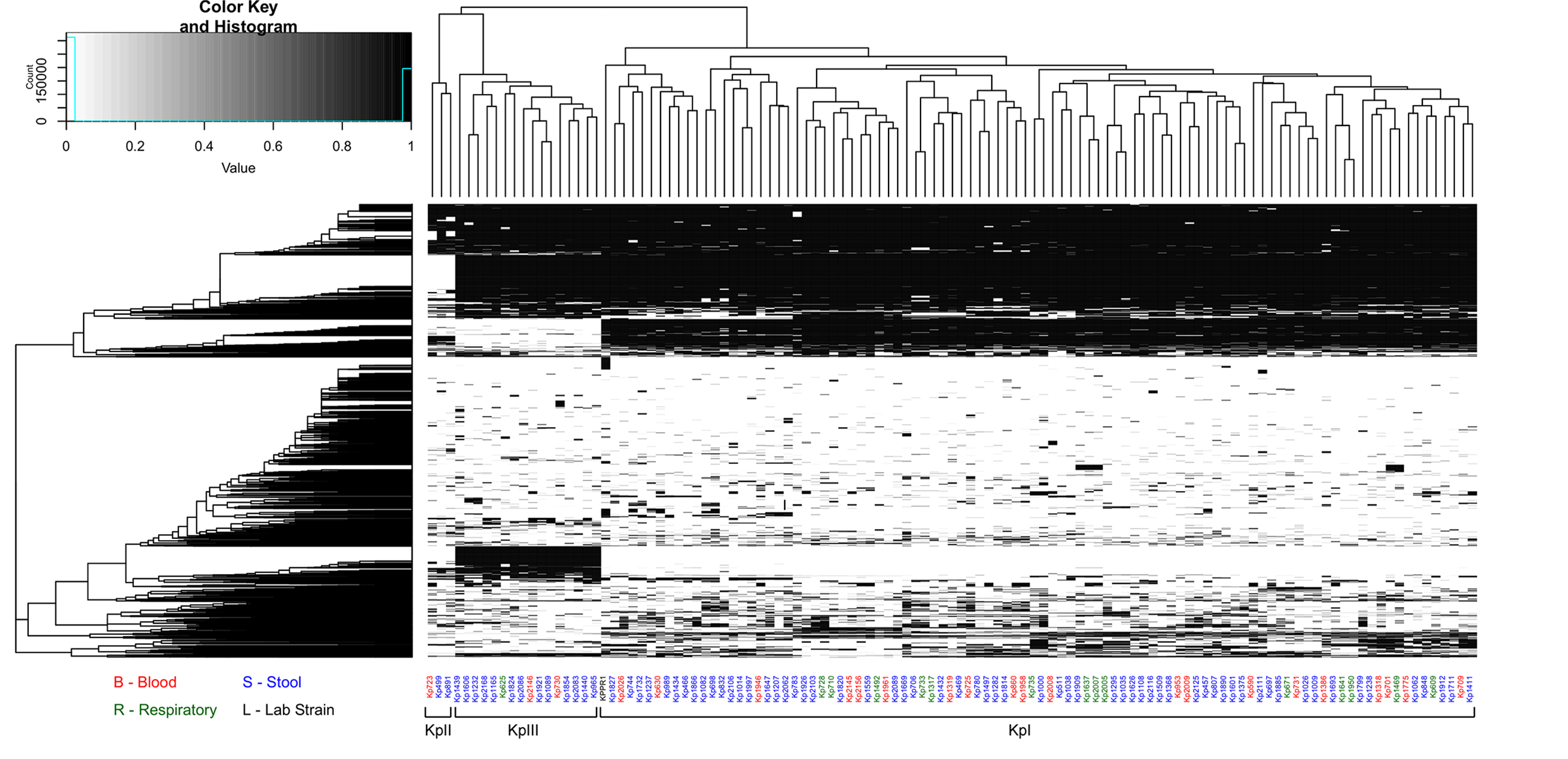

Supplement: FIG S4 [file sys003182238sf4.tif]

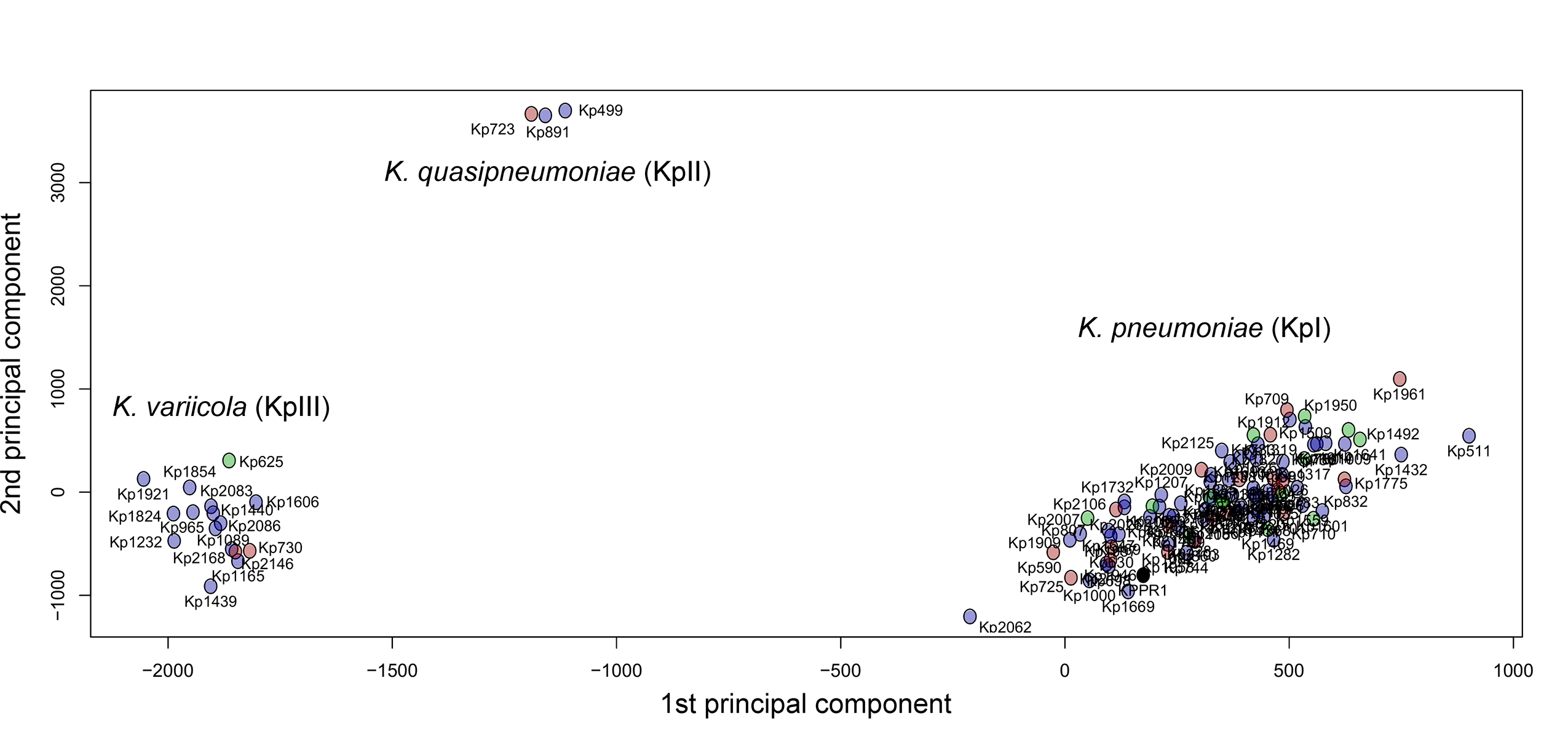

Supplement: FIG S5 [file sys003182238sf5.tif]

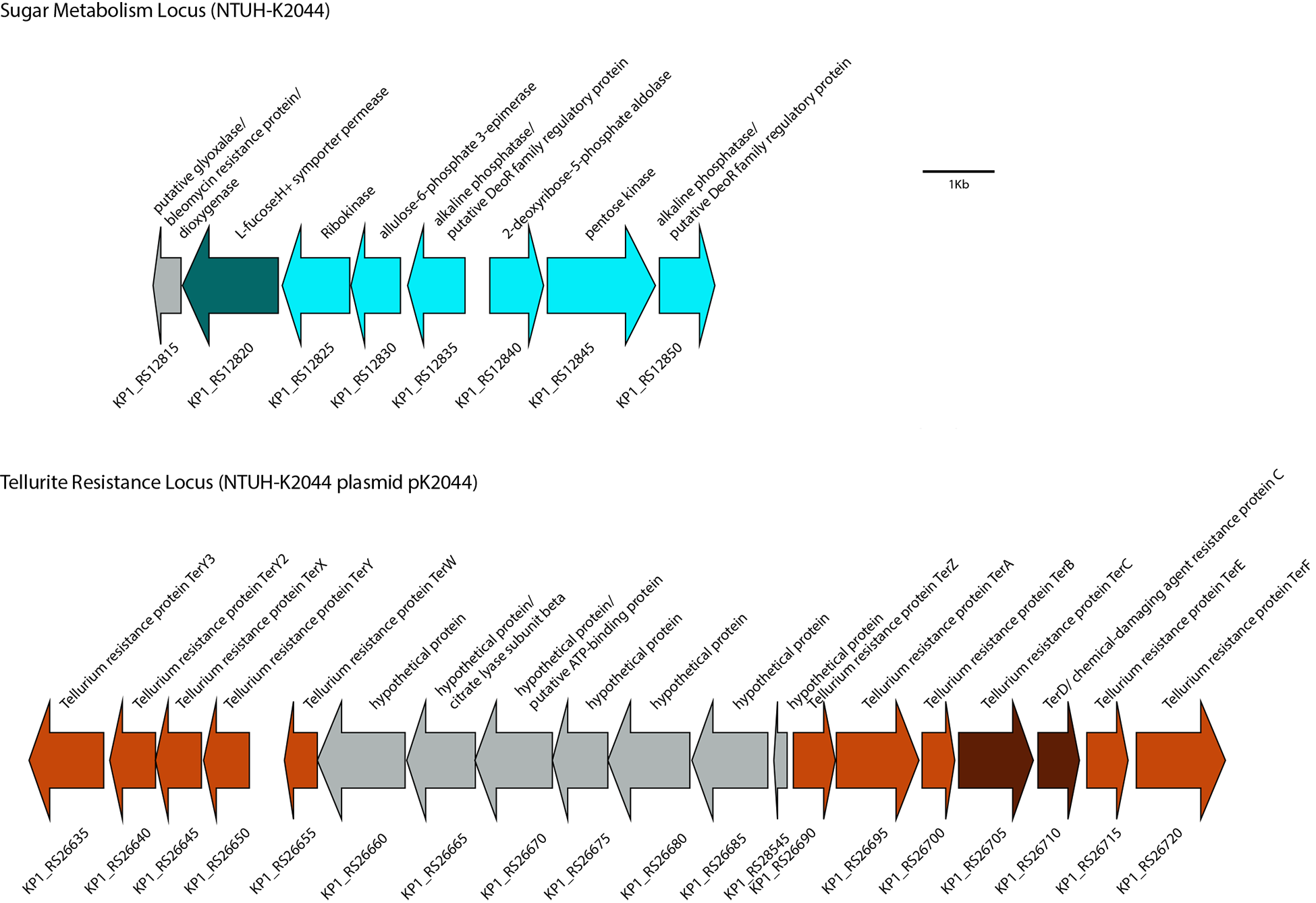

Supplement: FIG S6 [file sys003182238sf6.tif]

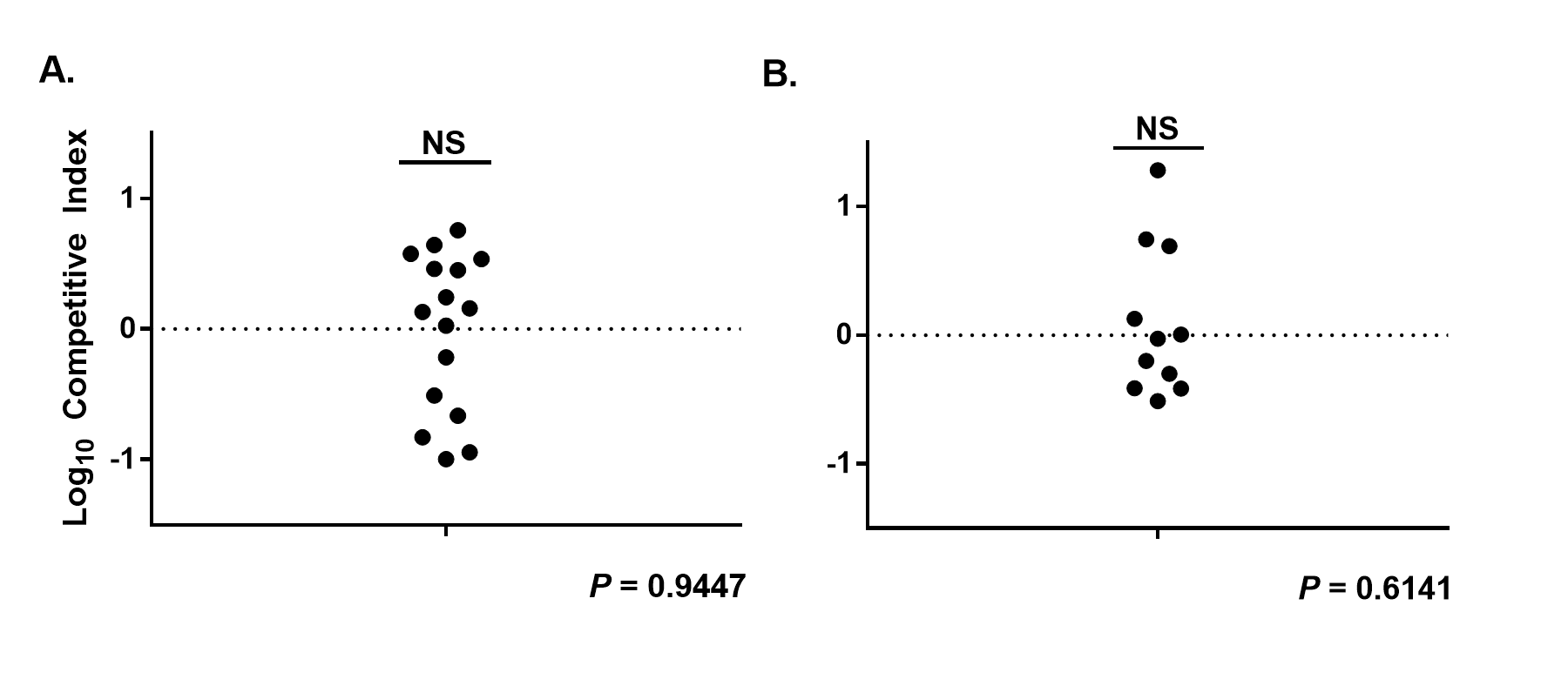

Supplement: FIG S7 [file sys003182238sf7.tif]
