# Supplementary material for: Identification of Pathogenicity-Associated Loci in Klebsiella pneumoniae from Hospitalized Patients
Source: mSystems. 2018 Jun 26;3(3):e00015-18. doi: 10.1128/mSystems.00015-18 (PMC6020474; doi:10.1128/mSystems.00015-18)
Supplement: TABLE S1 [file sys003182238st1.docx]

**Table S1.** **Multivariable model for psicose utilization locus association with infection.**

| **Variable** | **Odds Ratio** | **95% CI** | ***P* value** |
| --- | --- | --- | --- |
| **Psicose utilization locus** | 11.73 | 1.25-110 | .031 |
| **Fluid & Electrolyte Disorders** | 2.95 | 0.82-10.6 | .098 |
| ***K. pneumoniae* species** | 1.22 | 0.05-30.5 | .903 |
| **Minimum Serum Glucose (mg/dL)** | 1.04 | 1.01-1.08 | .012 |
| **Body Mass Index Baseline (kg/m^2^)** | 0.83 | 0.72-0.97 | .015 |
| **White Race** | 0.09 | 0.01-0.64 | .016 |
| **Peripheral Vascular Disease** | 0.02 | <0.01-1.01 | .051 |
