# Supplementary material for: Identification of Pathogenicity-Associated Loci in Klebsiella pneumoniae from Hospitalized Patients
Source: mSystems. 2018 Jun 26;3(3):e00015-18. doi: 10.1128/mSystems.00015-18 (PMC6020474; doi:10.1128/mSystems.00015-18)
Supplement: TEXT S1 [file sys003182238s1.docx]

Supplemental Materials and Methods:

*Bacterial identification and growth conditions*

Rectal swabs were collected during the course of clinical care (upon unit admission, weekly, and at discharge) and were transported and stored in the ESwab Transport System (BD, Franklin Lakes, NJ) at room temperature. Within 24 hours of receipt, 1μl of inoculated ESwab media was plated to MacConkey agar (Remel, Lenexa, KS) streaked for quantification and incubated for 18–24 hours at 35^o^C. The expected analytical sensitivity is 10^3^ CFU/mL of ESwab media. For each sample, three mucoid lactose fermenting (MLF) colonies were isolated as potential *K. pneumoniae* and subcultured onto blood agar plates (BAP) (Remel, Lenexa, KS) (1). If fewer than three MLF colonies were present in a particular sample, all were subcultured. The predominant morphotype was used for sequence analysis. Bacterial identification was performed using matrix-assisted laser desorption/ionization time of flight (MALDI-TOF). Isolates were stored at −80^o^C in Luria-Bertani (LB) Broth containing 20% glycerol, and were grown on either BAP or LB plates at 30^o^C overnight unless otherwise indicated.

*Whole genome sequencing*

Bacterial genomic DNA (gDNA) was isolated using the PUREGENE ® DNA Purification Kit (PUREGENE®, Minneapolis, MN). Purified gDNA was sent to the University of Michigan DNA Sequencing Core where it was sheared (200bp) and prepared as a multiplex library with unique barcodes for each sample. Whole genome sequencing was performed using the HiSeq 4000 sequencing system (Illumina, San Diego, CA).

*Pathogenicity-associated locus sequencing*

A reference sequence was created by combining the chromosomal and plasmid sequences of the five studied *Klebsiella* strains. A GFF file was created to include all gene annotations from these five strains. In generating the reference sequence, we kept all orthologous genes present in order to account for potential allelic differences amongst our sample isolates. The Burrows–Wheeler short-read aligner (BWA, version 0.7.5a-r405) was used to align the reads in its default mode (2). For 50 nucleotide reads, it allows up to three mismatches, and it outputs up to one optimal alignment for each read. If multiple optimal matches are found, it reports one randomly. The number of alignments that fell within each gene was counted in each sample, by intersecting the alignment with the pan-genome GFF file using BEDTools (3). The normalized read count values were calculated as raw counts divided by the number (in million) of dedupped alignments in that sample, and then by the corresponding gene length (in kb). Because we concatenated closely related *Klebsiella* strains, we expect to have duplicates of identical sequences due to homologous genes, and therefore random alignment among these genes. Orthologous genes were identified by comparing the genes from the five strains using CloVR Comparative (4). The genes that matched each other as orthologs, based on conservation of both nucleotide sequence and genome location, were grouped together to create a gene-bin. The normalized alignment count for each gene in a gene-bin was summed up to represent the coverage of this bin. In each sample, the normalized count sum values from all bins display a bimodal distribution. A k-means clustering approach (5) was applied to binarize the data into “0” or “1” indicating “absence” or “presence” of genes respectively.

*Multilocus sequence typing (MLST) and phylogenetic tree*

Raw sequencing reads (Illumina single end reads) for patient isolates, as well as FASTA files from GenBank for each reference isolate, were run through the Center for Genomic Epidemiology MLST typing scheme (https://cge.cbs.dtu.dk/services/MLST/). KpI, KpII, and KpIII group isolate sequence types were obtained from Maatallah et al (6), and MLST sequences for each Kp group isolate were obtained using <http://bigsdb.web.pasteur.fr>. Resulting sequences for each of the seven genes were concatenated for each isolate. Concatenated MLST sequences were aligned using ClustalX 2.1. The phylogenetic tree was constructed using MEGA6 (7) based on the neighbor-joining method (500 bootstrap replicates) and Jukes-Cantor distance. The tree was then uploaded into the Interactive Tree of Life database for annotation (<http://itol.embl.de/>) (8). Gene presence or absence was determined using the Normalized Sum Counts from our PAL-Seq data.

*Single nucleotide variant identification*

Quality of raw reads was assessed with Fastqc (9), and Trimmomatic (10) was used for trimming adapter sequences and low quality bases. Variants were identified by: 1) mapping filtered reads to the finished KPNIH1 reference genome (GenBank accession no. CP008827) using BWA, 2) discarding PCR duplicates with Picard, and 3) calling variants with SAMtools and bcftools. Variants were filtered from raw results using GATK’s VariantFiltration (QUAL > 100, MQ > 50, > 10 reads supporting variant, FQ <0.025). In addition, a custom python script was used to filter out single nucleotide variants that were: 1) <5 bp in proximity to indels 2) <10 bp in proximity to another variant, or 3) not present in the core genome. Lastly, for phylogenetic analyses, Gubbins was applied to filter out recombinant variants (11).

*WGS phylogenetic analysis*

Maximum likelihood trees were constructed in RAxML (12) wherein variants were modeled with a general-time reversible model. Bootstrap analysis was performed with the number of bootstrap replicates determined using the bootstrap convergence test and the autoMRE convergence criteria (-N autoMRE). Bootstrap support values were overlaid on the best scoring tree identified during rapid bootstrap analysis (-f a).

*Variant analysis of mutant strains*

Genomic DNA from NTUH-K2044, *ΔKP1_RS12820*, and *ΔterC* was purified using the DNeasy Blood and Tissue kit (QIAGEN Inc., Germantown, MD). DNA was sequenced using the Illumina NexteraXT kit on the Illumina MiSeq using a 2x250bp V2 kit. Whole genome sequences were assembled using the Genome Assembly service from PATRIC ([www.patricbrc.org](http://www.patricbrc.org)). Sequence variants were identified using the PATRIC Variation Analysis service. The NTUH-K2044 genome from GenBank was used as a reference genome.

**Supplemental References:**

1. Lidin-Janson G, Kaijser B, Lincoln K, Olling S, Wedel H. 1978. The homogeneity of the faecal coliform flora of normal school-girls, characterized by serological and biochemical properties. Med Microbiol Immunol 164:247-53.

2. Li H, Durbin R. 2009. Fast and accurate short read alignment with Burrows–Wheeler transform. Bioinformatics 25:1754-1760.

3. Quinlan AR, Hall IM. 2010. BEDTools: a flexible suite of utilities for comparing genomic features. Bioinformatics 26:841-842.

4. Agrawal S, Arze C, Adkins RS, Crabtree J, Riley D, Vangala M, Galens K, Fraser CM, Tettelin H, White O, Angiuoli SV, Mahurkar A, Fricke WF. 2017. CloVR-Comparative: automated, cloud-enabled comparative microbial genome sequence analysis pipeline. BMC Genomics 18:332.

5. Lloyd S. 1982. Least squares quantization in PCM. IEEE Transactions on Information Theory 28:129-137.

6. Maatallah M, Vading M, Kabir MH, Bakhrouf A, Kalin M, Nauclér P, Brisse S, Giske CG. 2014. *Klebsiella variicola* Is a Frequent Cause of Bloodstream Infection in the Stockholm Area, and Associated with Higher Mortality Compared to *K. pneumoniae*. PLOS ONE 9:e113539.

7. Tamura K, Stecher G, Peterson D, Filipski A, Kumar S. 2013. MEGA6: Molecular Evolutionary Genetics Analysis Version 6.0. Mol Biol Evol 30:2725-2729.

8. Letunic I, Bork P. 2016. Interactive tree of life (iTOL) v3: an online tool for the display and annotation of phylogenetic and other trees. Nucleic Acids Res 44:W242-W245.

9. Andrews S. 2010. FastQC A Quality Control tool for High Throughput Sequence Data.

10. Bolger AM, Lohse M, Usadel B. 2014. Trimmomatic: A flexible trimmer for Illumina Sequence Data. Bioinformatics doi:10.1093/bioinformatics/btu170.

11. Croucher NJ, Page AJ, Connor TR, Delaney AJ, Keane JA, Bentley SD, Parkhill J, Harris SR. 2015. Rapid phylogenetic analysis of large samples of recombinant bacterial whole genome sequences using Gubbins. Nucleic Acids Res 43:e15.

12. Stamatakis A. 2014. RAxML version 8: a tool for phylogenetic analysis and post-analysis of large phylogenies. Bioinformatics 30:1312-3.
